# Supplementary material for: The effects of genital myiasis on the diversity of the vaginal microbiota in female Bactrian camels
Source: BMC Vet Res. 2022 Mar 5;18:87. doi: 10.1186/s12917-022-03189-5 (PMC8897907; doi:10.1186/s12917-022-03189-5)
Supplement: Supplementary file 5 — Additional file 5. [file 12917_2022_3189_MOESM5_ESM.zip › MPL201709200_16s_yy/Treat1/C07_picrust/KEGG_L2_Metabolism.pdf]

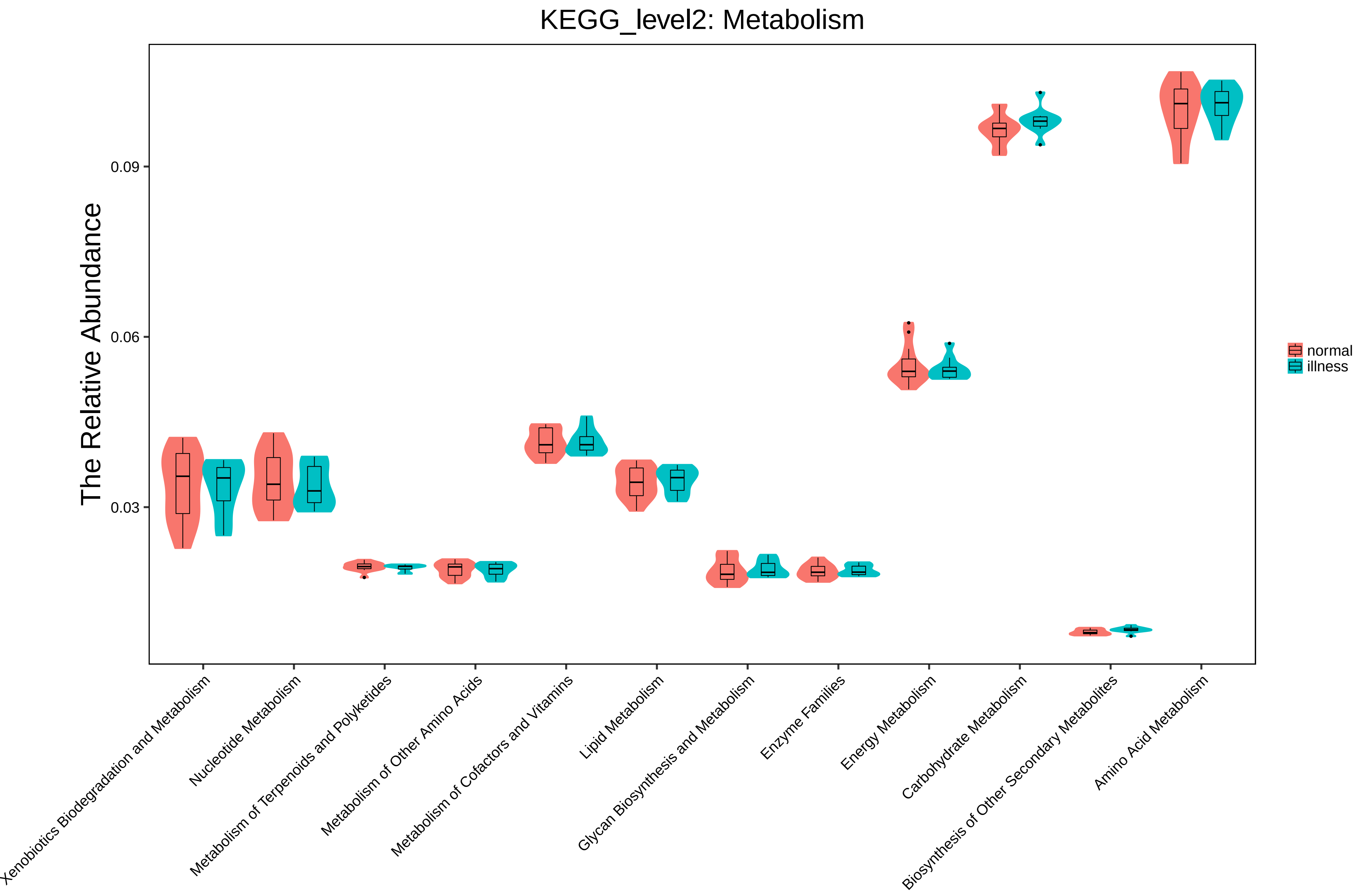
KEGG_level2: Metabolism
The Relative Abundance
0.09
0.06
0.03
normal
illness
Xenobiotics Biodegradation and Metabolism
Nucleotide Metabolism
Metabolism of Terpenoids and Polyketides
Metabolism of Other Amino Acids
Metabolism of Cofactors and Vitamins
Lipid Metabolism
Glycan Biosynthesis and Metabolism
Enzyme Families
Energy Metabolism
Carbohydrate Metabolism
Biosynthesis of Other Secondary Metabolites
Amino Acid Metabolism
